# Retrograde intramedullary nailing for femoral shaft fracture with ipsilateral dynamic hip screw fixation for femur neck fracture; dual fixation. Case study

**DOI:** 10.1016/j.ijscr.2025.112093

**Published:** 2025-10-25

**Authors:** Said Osman Dahir, Abdirahman Omer Ali, Mohamoud Hashi Abdi, Ridwan Mohamed Farah, Hodo Abdi Abdillahi, Hassan Elmi Moumin

**Affiliations:** a School of Medicine and Surgery, College of Health Sciences, Amoud University, Borama, Somalia; b School of Postgraduate Studies and Research, Amoud University, Amoud Valley, Borama, 25263, Somalia; c Borama Regional Hospital, Surgery Department, Borama, Somalia; d Borama Regional Hospital, Radiology Department, Borama, Somalia; e Hargeisa Group Hospital, Hargeisa, Somalia

**Keywords:** Femoral Shaft Fracture, Femoral Neck Fracture, Ipsilateral Fracture, case report

## Abstract

**Introduction:**

Ipsilateral femoral neck and shaft fractures are rare, high-energy injuries posing diagnostic and management challenges. Optimal treatment strategies remain debated. This case study presents a successful approach using dual fixation with retrograde intramedullary nailing and dynamic hip screw (DHS) fixation.

**Case presentation:**

A 38-year-old male driver presented following a motor vehicle accident with right thigh and hip pain, swelling, and deformity. Radiographic evaluation revealed a transverse femoral shaft fracture combined with an ipsilateral, non-displaced transcervical femoral neck fracture.

**Discussion:**

Dual fixation with retrograde femoral nailing for the shaft and DHS for the neck offers a stable construct while minimizing soft tissue disruption and potentially facilitating early weight-bearing. While open reduction and internal fixation can lead to complications, our chosen technique aimed to avoid them. Vigilant monitoring for complications like avascular necrosis, nonunion, and infection remains crucial. This case highlights the potential efficacy of dual fixation in achieving favorable outcomes in these complex fracture patterns.

**Conclusion:**

Dual fixation using retrograde intramedullary nailing for femoral shaft fracture and DHS fixation for the ipsilateral femoral neck fracture represents a viable treatment option, especially in non-displaced neck fractures. Further research with larger cohorts is needed to validate these findings and establish definitive treatment guidelines.

## Highlights

• Describes a 38-year-old male with ipsilateral femoral shaft and neck fractures treated with dual fixation
• Retrograde intramedullary nailing of the shaft and DHS fixation of the neck achieved stable fixation.
• The case suggests dual fixation minimizes soft tissue disruption and may promote early mobilization.
• Provides a treatment approach for this challenging combination of fractures, especially with non-displaced neck fractures

## 1 Introduction

Ipsilateral femoral neck and shaft fractures are relatively rare yet present significant clinical difficulties, accounting for approximately 2.5–9 % of all femoral fractures. This distinct fracture variant was documented for the first time in 1953 [1,2]. The concomitant fractures of the femoral neck and shaft typically arise from high-energy traumas, such as motor vehicle collisions (MVC) and falls from significant elevations. The fracture manifests when the hip experiences axial loading concurrent with leg abduction [3,4]. Affected individuals are predominantly young and frequently present with multiple concomitant injuries [5]. The identification of the femoral neck fracture is often postponed in 19–31 % of patients [6]. The management of ipsilateral femoral neck and shaft fractures presents considerable complexity, and a multitude of protocols exist for their treatment. The potential therapeutic approaches include: (1) antegrade femoral nailing of the shaft, supplemented by cancellous screws located anterior to the nail for the stabilization of the neck [6]; (2) reconstruction-type intramedullary nailing [1,7]; (3) various combinations of plate constructs [which may involve a dynamic hip screw (DHS) paired with a long side plate configuration, a hip screw accompanied by a short side plate for the neck, and a separate plate for the shaft, or the utilization of cancellous screws for the femoral neck alongside a plate for the shaft] [8], (4) retrograde intramedullary nailing of the shaft combined with screw fixation of the neck [8]. The three predominant concerns associated with these fractures pertain to the optimal timing of surgical intervention, the sequence of fracture stabilization, and the selection of the most effective implant to employ [8]. This report is written in line with the SCARE 2025 guideline [9].

## 2 Case presentation

A 38-year-old male driver presented following a motor vehicle accident with right thigh and hip pain, swelling, and deformity. The patient has no significant past medical or surgical history and no relevant family history of orthopedic or significant medical conditions. He reported no current medications. Radiographic evaluation revealed a transverse femoral shaft fracture combined with an ipsilateral, non-displaced transcervical femoral neck fracture.

## 3 Examination

On initial examination, the patient was alert but appeared to be in significant pain. His vital signs were stable. Musculoskeletal examination revealed significant swelling, deformity, and shortening of the right thigh. Distal neurovascular examination of the right lower extremity was intact. No open wounds were present.

## 4 Investigations

**Fig. 1:**
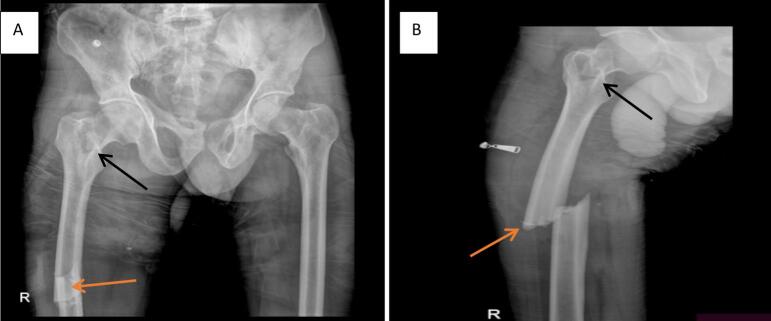
AP (A) and Lateral (B) X-ray views demonstrating a complete transverse femoral shaft fracture with shortening and complete displacement (orange arrows). A lucency consistent with a non-displaced transcervical femoral neck fracture is also visible (black arrows).

**Fig. 2:**
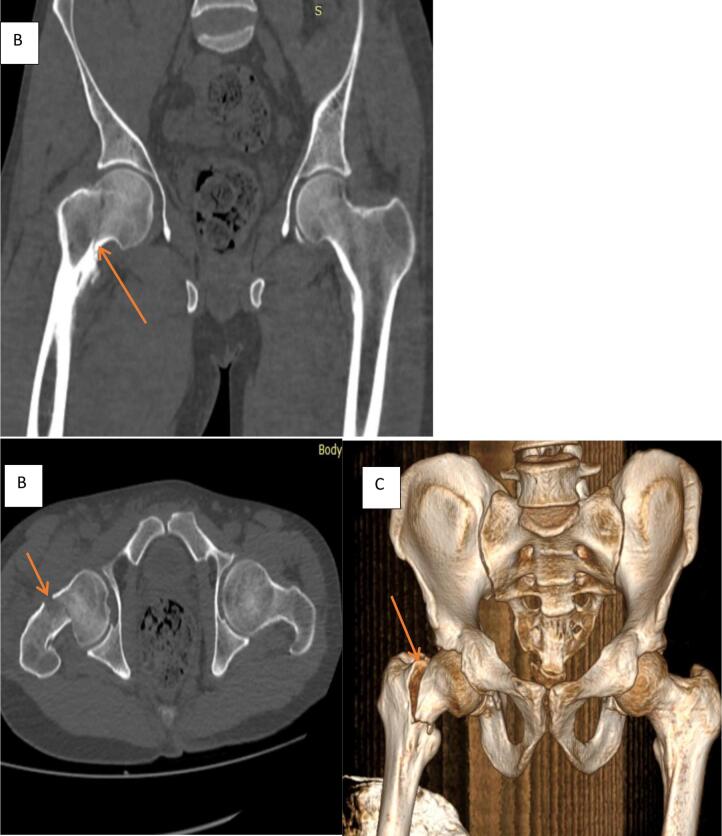
Pelvic CT Scan coronal and axial 3D rendering image demonstrating non-displaced trans cervical right femoral neck fracture orange arrows in A, B and C.

Following Advanced Trauma Life Support (ATLS) protocols [4], initial resuscitation and analgesia were administered. A radiographic evaluation was performed. A plain radiograph of the right femur revealed a transverse fracture of the femoral shaft (Fig. 1). Subsequent computed tomography (CT) scan of the pelvis revealed an ipsilateral, non-displaced transcervical fracture of the femoral neck (Fig. 2).

## 5 Diagnosis

Based on the clinical and radiographic findings, the patient was diagnosed with a right femoral shaft transverse fracture with an ipsilateral femoral neck transcervical non-displaced fracture.

## 6 Treatment

Following the diagnosis, the patient was admitted to the orthopedic ward for surgical management. The surgical plan involved dual fixation: retrograde femoral nailing for the femoral shaft fracture and dynamic hip screw (DHS) fixation for the femoral neck fracture.

## 7 Surgical technique

**Fig. 3:**
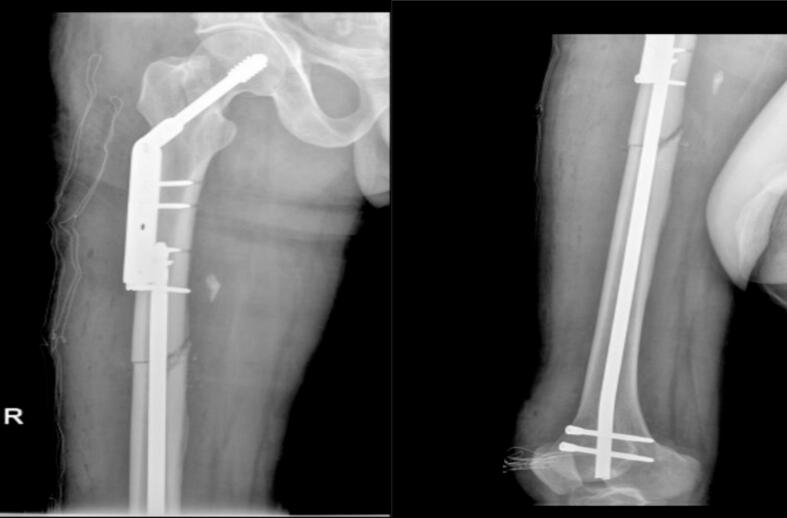
Retrograde intramedullary nail and the femoral neck fracture was reduced anatomically and fixed with a dynamic hip screw (DHS) system.

The patient was placed supine on an operating table. A retrograde intramedullary nail was inserted through the distal femur intercondylar notch on the medial side of the lateral condyle as the entry point through a lateral knee arthrotomy. The fracture was reduced and stabilized with an intramedullary SIGN nail with interlocking distal and proximal screws. Fluoroscopic imaging was utilized throughout the procedure to confirm fracture reduction and implant placement (Fig. 3).

## 8 Postoperative course

The patient tolerated the procedure well and was transferred to the post-anesthesia care unit in stable condition. Postoperative pain management was initiated with a multimodal analgesic regimen. Thromboprophylaxis with low-molecular-weight heparin was administered to prevent deep vein thrombosis (DVT) [10]. Six weeks of non-weight-bearing on the right lower extremity were encouraged. A structured physiotherapy and rehabilitation program was initiated to improve range of motion, muscle strength, and functional recovery. The patient was closely monitored for potential complications, including avascular necrosis (AVN) of the femoral head, nonunion, malunion, infection, and hardware failure. Serial radiographs were obtained to assess fracture healing.

## 9 Discussion

In this case study, we successfully treated a 38-year-old male with an ipsilateral femoral neck and shaft fracture using a dual fixation approach: retrograde intramedullary nailing for the femoral shaft and dynamic hip screw (DHS) fixation for the non-displaced transcervical femoral neck fracture. This technique provided a stable construct, facilitated early mobilization, and avoided complications typically associated with more invasive open reduction methods, leading to a favorable early clinical outcome.

Ipsilateral femoral shaft and neck fractures constitute rare injuries, primarily resulting from high-energy trauma [11,12]. The mechanism of injury typically involves a combination of axial loading, bending, and rotational forces [[12], [13], [14]]. Diagnosing these fractures poses a significant challenge, particularly in scenarios where one fracture obscures the other. Thus, maintaining a heightened index of suspicion and conducting a thorough radiographic assessment, which should encompass both conventional radiographs and computed tomography scans, is imperative to prevent overlooked injuries [2,7,8,17].

The management of ipsilateral femoral shaft and neck fractures is notably intricate [18]. The treatment of these fractures necessitates a nuanced approach that considers multiple variables, including the fracture configuration, the age of the patient, physiological condition, and the experience level of the surgeon [8]. Numerous fixation methodologies have been documented, such as open reduction and internal fixation (ORIF) utilizing plates and screws, cephalomedullary nailing, and dual fixation [[17], [18], [19]]. Open reduction accompanied by plating for femoral shaft fractures has been associated with a heightened incidence of complications such as infection and nonunion [20]. In the present case, we opted to implement dual fixation utilizing retrograde femoral nailing for the shaft fracture in conjunction with dynamic hip screw (DHS) fixation for the neck fracture. Retrograde nailing provides several benefits, including minimal disruption of soft tissues, a decreased likelihood of nonunion, and the facilitation of early weight-bearing [21]. DHS fixation is a well-established modality for addressing femoral neck fractures, ensuring stable fixation while permitting controlled impaction at the fracture site [12,15,22]. The non-displaced character of the femoral neck fracture rendered it suitable for DHS fixation. Potential complications linked with these fractures encompass avascular necrosis (AVN) of the femoral head, nonunion, malunion, infection, hardware failure, and thromboembolic phenomena [17,18,27]. Vigilant observation and strict adherence to established protocols for thromboprophylaxis and infection prevention are vital to mitigate these risks. Extended follow-up is critical to evaluate the healing of the fracture, functional outcomes, and the emergence of any late complications.

## 10 Conclusion

The management of ipsilateral femoral neck and shaft fractures remains challenging. This case study supports the use of dual fixation with retrograde intramedullary nailing and dynamic hip screw fixation as a potential solution. While our results are encouraging, future research should focus on comparing this technique to other methods, identifying ideal patient selection criteria, and defining long-term outcomes to establish evidence-based guidelines for the optimal treatment of these rare and complex fractures.

## Author contribution

Dr. Said Osman Dahir, Dr. Abdirahman Omer Ali, Dr. Ridwan Mohamed Farah and Hassan Elmi Moumin contributed in taking history and providing care to the patient throughout his hospital stay. Additionally, Dr Abdirahman Omer Ali, Hassan Elmi Moumin and Dr. Said Osman Dahira contributed to the development of the manuscript. Dr. Mohamoud Hashi Abdi is the radiologist.

## Consent for publication statement

Written informed consent was obtained from the patient for publication and any accompanying images. A copy of the written consent is available for review by the Editor-in-Chief of this journal on request.

## Ethical approval

The study protocol, case investigation, and consent form were thoroughly examined by the institutional review board of the College of Health Sciences at Amoud University. They granted approval for the study, along with the Ministry of Health and Borama Regional Hospital in Awdal Region, Somaliland (BRHH-220/2024). Prior to participation, written informed consent was obtained from every individual involved.

## Guarantor

Dr.Hassan Elmi Moumin, on behalf of all authors, accept full responsibility for the work

## Research registration number

Number Not applicable.

## Funding

The study did not receive funding.

## Conflict of interest statement

The authors report no declarations of interest.
